# Supplementary material for: The billion-dollar case for sustaining palaeontology’s digital databases
Source: Nat Ecol Evol. 2026 Feb 10;10(3):594–605. doi: 10.1038/s41559-026-02985-8 (PMC12971485; doi:10.1038/s41559-026-02985-8)
Supplement: Supplementary file 1 — Supplementary Tables 1–8, Methods and Discussion in the following sections: (1) stable links to extended methods, code and extended data; (2) glossary of terms; (3) volunteer survey process; (4) benefit framework of open science (publication products); (5) database survey, protocol, financial valuation and analysis; (6) relevant links; and (7) citations. [file 41559_2026_2985_MOESM1_ESM.pdf]

nature ecology & evolution

Supplementary information https://doi.org/10.1038/s41559-026-02985-8

The billion-dollar case for sustaining palaeontology's digital databases

In the format provided by the authors and unedited

Contents

1. Data and code links
2. Glossary
3. Volunteer survey of the authorship: Database maintainers, curators, and data contributors
4. Benefits of Open Science in Earth Science
5. Database Survey
6. Relevant Links
7. Citations

1. Data and code links

Stable links:

CodeOcean: Dowding, E. M., et al. Fossils for Future: the billion dollar case for palaeontology's digital infrastructure. [Codebase] CodeOcean. (2025) https://doi.org/10.24433/CO.1586965.v1
Zenodo: Dowding, E. M., et al. Fossils for Future and the billion dollar case for palaeontology's digital infrastructure. [Dataset] Zenodo. (2025). https://doi.org/10.5281/zenodo.17828000

GIT:
Parent https://github.com/dowdingem/IRAL/tree/main

Author survey and financial valuation: CuratorReview
https://github.com/dowdingem/IRAL/blob/main/Data/CuratorReview.xlsx

Database survey
https://github.com/dowdingem/IRAL/blob/main/Data/IRAL.csv

Database use: Publication products
https://github.com/dowdingem/IRAL/blob/main/Data/Paleo%20database%20publication%20products.xlsx

Code
https://github.com/dowdingem/IRAL/blob/main/IRAL1707.R

2. Glossary

Supplementary Table 1: Glossary of specific terms used in this article that relate to paleontological and Earth Science databases.

These terms are internally consistent with the main text of Dowding et al and are altered from the CODATA Research Data Management Terminology [https://vocabs.ardc.edu.au/viewById/685] for discussion of palaeontological use cases. Terms here are not considered definitive.

**Community-developed or community-run resources**	Scientific data resources that are built, curated, and maintained by multi-institutional teams of researchers and associates. Here, the Earth Science and paleontological international research community.
**Data coverage**	The completeness, connectedness, and relevance of data stored in a database or data system. This term comprises Sample and Entity coverage
**Database**	A structured system that stores datasets and the relationships within and among datasets and their metadata. Databases are dynamic and regularly updated as datasets are added or curated.
**Dataset**	An arbitrarily defined single set of structured or semi-structured data, e.g. the taxonomic identities and abundance information for fossil occurrences recovered from one or more sites. Datasets tend to be static once created, with occasional updates, e.g. to refine taxonomic identities or age inferences.
**Data platform**	The software services built around a database, usually consisting of a layered stack of software interfaces for backend database management, data entry and curation, data exploration and visualization, and statistical analysis and modeling.
**Data system**	A set of records collected by a research community, and the accompanying analytical and conceptual frameworks for interpreting these measurements; a data system usually comprises many data types (after Farley et al, 2018). They can extend to an interacting set of data platforms and their networks of data contributors, curators, and users. Data systems can vary in the degree of internal linkages and interoperability.
**Data provenance**	The ability to trace back any given digital object through all stages of analysis back to its original collection event (sampling, observation, specimen acquisition), used to ensure authenticity, to support reproducibility, and to give proper credit for scientific contributions at all stages of effort .
**Entity**	A discrete digital datum that represents some facet of information about a paleobiological object.
**Entity coverage**	The database's ability to accommodate a wide range of data types and link them through compatible internal connections. A subset of Data coverage.
**Fundamental data**	The observation, sampling, and recording of the sedimentary record, assemblage data, and fossil specimens within these sediments with little or no interpretation. Examples include geochemical or trait measurements on fossil specimens, multimedia recording, counts of fossil specimens, stratigraphic position, or geospatial locations. Also called primary data. A counterpart to Processed data.

**Processed data**	The interpretive information generated from the fundamental data, whether by inference or deduction. Can include paleoenvironmental analysis, taxonomic identification, paleoclimate, paleolocations, sea level, etc.
**Sample**	A representative part from a larger whole or group (e.g. a population). Can become a specimen for a particular scientific use.
**Sample coverage**	Representative and relevant collection of samples (sampling) to address a given question.
**Specimen**	A physical object from which data are collected (morphotraits, isotope measurements, assemblage, taxon id, etc); material sample, evidence, multimedia, etc.

3. Volunteer survey of the authorship: Database maintainers, curators, and data contributors

The authors of this paper, who were also database maintainers and/or developers, volunteered information about the back-end, data volume, and support structures. These descriptions informed recommendations and present a clear synthesis of the variability in database structure and maintenance. The provided database ages were incorporated into the database survey.

FILE NAME: CuratorReview.xlsx

Supplementary Table 2*: A list of the paleontological and earth science databases represented by the authors of this article, including their year of inception, governance type, specialisation and scopes. Except for FRED, all listed databases are global in scope, but sampling is heterogeneous (e.g. some data types in NEOTOMA have a higher sampling density in North America, while the GBDB has a higher sampling density in China). OA = Open Access, CC = Community curated, Museum = government/ geoscience survey hosted and/or state funded. Highlighted GREEN indicate active but recent databases that are addressed for boundary conditions in time series.*

Database name	Inception	Governance Type	Specialisation and scope	
			Temporal	Taxonomic
**Neptune Sandbox Berlin (NSB)**	1989	OA, CC	Jurassic to Recent	Planktonic protists

https://www.museumfuernaturkunde.berlin/en/research/nsb-database				
**PaleoReefs Database PARED**	1995	OA, CC	Ediacaran to Quaternary	Fossilreefs and their attributes
https://www.paleo-reefs.pal.uni-erlangen.de/				
**New and Old Worlds database (NOW)**	1996	OA, CC	Cenozoic	Mammals
https://nowdatabase.org/				
**Paleobiology Database (*PBDB)***	1998	OA, CC	Geological time	All life
https://paleobiodb.org				
**NHNM Paleobiology**	2001	Museum	Geological time	All life
https://collections.nmnh.si.edu/search/paleo/				
**The Fossil Record Electronic Database (*FRED)***	2003	Museum	Geological time	All life (in NZ)
https://fred.org.nz/				
**Macrostrat**	2005	OA, CC	Geological time	All life (biostratigraphic focus)
https://macrostrat.org/				
**Geobiodiversity database (*GBDB)***	2006	OA, CC	Ediacaran to Quaternary	All life
http://www.geobiodiversity.com/home				
**Neotoma Paleoecology database (Neotoma)**	2007	OA, CC	Geological time	All life and stable isotopes
https://www.neotomadb.org/				

**Extending Ocean Drilling Pursuits (eODP)**	2019	In dev, CC	Jurassic to recent	All life
https://eodp.github.io/index.html				
**Ancient Reef Traits (ART)**	2020	OA, CC	Mesozoic - Recent	Reef builders
https://art.nat.fau.de/				
**Triton**	2021	OA, CC	Cenozoic	Planktonic foraminifera
https://www.nature.com/articles/s41597-021-00942-7 // https://doi.org/10.6084/m9.figshare.14655564				
**BioDeepTime (BDT)**	2023	OA, CC	Ordovician to recent	All life
https://biodeeptime.github.io/				
**Phenotypic Evolution Time Series (PETS) database**	2023	OA, CC	Cambrian to recent	Metazoan life
https://pets.nhm.uio.no/				
**Biotic Interactions in Deep Time (*BITE*)**	2024 -In Dev	In development, CC	Ediacaran to recent	All life

4.Benefits of Open Science in Earth Science

To highlight the value to the scientific community, a survey of the literature that cites 6 databases was conducted in 2024 (>2000 articles). The number of these articles was counted, and the author team assigned topic tags based on the title, keywords, and abstract context to assess the variety of topics that paleontological and Earth science databases were used for.

Topic tags were: paleobiogeography, diversity, taxonomy, morphology, phylogeny, paleoecology, environment, taphonomy, paleoclimate, conservation, geochemistry, sedimentology, stratigraphy, evolution, and other.

The database data was used across each topic, with ‘other’ consistently ranking in the top 3 of tags (except the PBDB), suggesting that the nominated tags did not capture the diversity of topics that palaeontological databases contributed to.

FILE NAME: Paleo database publication products.xlsx

In support of the aims of Open Access databases and relevant for table 3 in the main text, we use this information to describe the benefits of Open Science, altering the NSF (2018) framework for a palaeontological and Earth Science database community.

Supplementary Table 3. *Benefits of Open Science for palaeontology and Earth Sciences, focusing on open data, based on NSF (2018) and the FAIR (Jacobsen et al., 2020), relevant for table 3 main text.*

Benefit	Description
**1. Rigor and reliability**	Easier reporting, greater reproducibility, publicity of demonstrable soundness and validity.
**2. Ability to address new questions**	Interdisciplinary collaboration can bridge the gap between data availability and expertise to propose and address new research avenues.
**3. Faster and more inclusive dissemination of knowledge**	Open Access makes the results of scientific research more available, attainable, and mitigates inequality. Scientific efforts are bolstered in impact and reach, accelerating scientific discourse by removing paywall barriers.
**4. Broader participation in research**	Increases broad engagement with science as a ‘reader’ of the discourse and application to external groups, e.g. in education, public policy, etc. Further increases citizen science and career pathways into paleo- and geoscience.
**5. Effective use of resources**	Retains and consolidates the research, development, and funding resources poured into paleo-data resources. Modular, open data systems would ease data integration and aggregation across data types and between fields. Facilitates greater ease of analysis with less redundancy and waste.
**6. Improved performance of research tasks**	Open data, code, workflows, and repositories facilitate the development of processes and reduce repeated effort. Maintaining stable version-controlled data products, such as paleoclimate models, ensures reproducible and transparent results as databases, software, and packages are updated.

**7. Open publication for public benefit**	Many fields (for example, education, public policy, the arts and humanities, and industry) all benefit from access to paleoscience resources. Increasing access increases engagement, leading to increased perception of paleo-data's value and application, developing new knowledge and products.

5.Database survey

To assess the temporal dynamics and sustainability of palaeontological databases, we recorded key data related to each database's lifespan and accessibility. Between November 2024 and March 2025, aggregators (Web of Science and Google Scholar) were queried for multilingual instances of 'Database' (Table 1) and 'Palaeontology', 'Geology', 'Fossil', 'Earth Science', and combinations thereof.

FILE NAME: IRAL.CSV

Supplementary Table 4: *List of core search terms used for web search*

Term	Language
Earth Science database	English
Base de datos de ciencias de la tierra	Spanish
Base de données des sciences de la Terre	French
قاعدة بيانات علوم الأرض	Arabic
Fossil Database	English
Base de datos de fósiles	Spanish
Base de données de fossiles	French
قاعدة البيانات الأحفورية	Arabic
Geology database	English

Base de datos de geología	Spanish
base de données géologique	French
قاعدة البيانات الجيولوجية	Arabic
Palaeontology database	English
Paleontology database	North American English
Base de datos de paleontólogos	Spanish
Base de données de paléontologie	French
قاعدة بيانات علم الحفريات	Arabic

Web of Science search was conducted within the *Physical, Chemical & Earth Sciences*, and *Life Sciences* categories in all listed languages.

Languages were chosen as the four most spoken languages by distribution: English, Spanish, Arabic, and French. Distribution was decided by the number of official or co-official languages by country counts (from the South Australian Government, 2017). Other major languages by speaker population were searched, for example, Mandarin, for more than 5 pages. If no new/novel non-domestic government databases were identified, the search was ended.

The first 10 result pages were inspected for each aggregator (100 results on Google Scholar; 250 for Web of Science), where each result link was opened and the result read. The presentation of new databases, as opposed to results returning the use of a database, were recorded. Key definitions for the fields in Table 2 are below.

Supplementary Table 5: *Recorded fields of database survey*

Term	Definition
**Database name**	Name of the database.
**Data description**	Collation focus of the database, e.g. fossil mammal occurrences and/or isotopic records. Recorded type, e.g. a relational database or a series of datasheets.

**Website**	Weblink to access point, e.g. API or data repository (when available).
**Inception**	Date of website release and/or publication.
**2024 Active**	Whether or not the database was both accessible and recently maintained.
**Last reference/update**	Latest available evidence of maintenance: e.g. website update, published versioning.
**Reference**	Citation (for relevant release or publication
**Integrated with other databases**	Whether the database is supported by another, e.g. the Neotoma constituent databases.
**Governmental**	Whether the database is developed and hosted by a government
**Generalist**	Whether the database had a broad focus in that the data description includes multiple higher-order taxa and/or datatypes? E.g. compare Neotoma (generalist) to Triton (specialist).
**Specialist**	Whether the database had a specific focus in that the data description includes one higher order taxa and/or datatypes? E.g. Triton.
**Funding**	Whether the reference includes funding information; when available the name of the funding and grant number was recorded in notes.
**Omit**	Databases that were developed or hosted by government agencies or commercial entities were excluded from the analysis.

5.1 Methods: Data Collection and Definitions

The procedural recording of information for the inception and last update were considered for the ‘start’ and ‘end’ dates of the database activity.

Expanded key terms and procedure:

Start Date: The start date was defined as the year the database was first made publicly available. This included the publication of associated journal articles or the launch of a dedicated website—whichever came first.

End Date: The end date was defined as the most recent year the database was known to be updated. This was typically taken from the update information available on the database's official website. In cases where such information was not available, we used the most recent publication or resource that documented the state of the database. If no such record existed, the end date was approximated by identifying the most recent

scientific publication that cited or used the database. Where no evidence of activity beyond initial publication could be found, the end date was recorded as the last year in which the database was confirmed to be publicly accessible.

Start and End Dates Identical: Databases with identical start and end dates were included in overall diversity metrics but excluded from range-through and extinction analyses, as they do not represent a temporal span of activity.

Funding Information: Information on funding was recorded when available, either from database websites or associated publications. In cases where no funding details could be confirmed—even after attempts to identify grant numbers or acknowledgements—this was noted as "No funding information available." For analysis, a simple Yes/No categorisation was used to indicate whether any funding data could be recovered.

This structured approach allows for consistent comparison across databases and ensures transparency in the handling of incomplete or inconsistent data.

When start or end dates were not available, the record was omitted.

5.2 Methods: Analysis

171 palaeontological and Earth science databases were identified, governmental were removed, retaining 125, of which 118 met the criteria (cleaned set available on GIT). For the summary statistics on database duration, same-year databases were omitted (range of zero), reducing the number considered to 88 databases.

118 were analysed in the figures removing the top ranking range.

Boundary effects at the beginning and end of the time series (e.g. high extinction rates in 2024-5) were mitigated by extending end dates into the future and pruning the time series at the end of 2023-24. Front boundary effects were mitigated by extending the beginning of the time series to include static databases, stretching the start point to the 1970s, before the proliferation of digital databases, which are the target group of the paper.

***Supplementary Table 6*:** *Top 15% of database longevity (longest duration of activity in years).*

**Name**	**Duration of activity (until 2024)**
Index to marine and Lacustrine Geological Samples	47
Palynodata	32
Neptune Sandbox Berlin	30

Latin America Pollen Database	30
Neogene Mammals of the old and new world	29
PaleoReefs Database	29
Mascot	26
Paleobiology Database	24
ION	24
World Foraminifera Database	23
Morphomank	23
Diatom paleolimnology Data Cooperative	23

Supplementary Table 7**:** *Summary Statistics of Database duration (years)*

	**Number**	**Mean**	**Median**	**Min**	**Max**	**Standard Deviation**	**Variance**
**Omitting Range of Zero**	88	11.3	9	1	47	9.4	87.9
**Bottom 85%**	75	8.4	7	1	21	6.1	37.7

5.3 Methods: Figures and additional metrics

Using rolling means of the raw numbers (including “range of zero” point occurrences), analysed using R studio and DivDyn (Kocsis et al, 2019).

1. **Diversity by duration (years active)**
2. **Richness by year**
3. **Rolling mean extinction rate by years active**
4. **Rolling mean origination by years**

Supplementary Table 8: *In-text metrics used to assess diversity dynamics modified from Kocsis et al (2019, table 2) and references therein.*

The following metrics were considered in both raw and rolling mean treatments for origination, extinction, and diversity. Result can be accessed by selecting the metric using divDyn (Kocisis et al, 2019). *** Indicates that the metric was used in the main figures

Variable name	Metric Name	Type
tSing, tOri, tExt, tThrough	Range-based taxon patterns	Counts
t2d, t2u, t3, tPart, tGFu, tGFd	Occurrence-based taxon patterns	Counts
txtProp, oriProp	Proportional extinctions and originations	Turnover
PC: extPC***, oriPC***	Per capita extinction and origination rates	Turnover
ext3t, ori3t	Three-timer extinction and origination rates	Turnover
extC3t, oriC3t	Corrected three-timer extinction and origination rates	Turnover
GF: extGF, oriGF	Gap-filler extinction and origination rates	Turnover
ext2f3, ori2f3	Second-for-third substitution extinction and origination rates	Turnover
divSIB***	Sampled-in-bin diversity (SIB)	Richness
divRT***	Range-through diversity (RT)	Richness
divBC	Boundary-crosser diversity (BC)	Richness
divCSIB	Corrected sampled-in-bin diversity	Richness

5.4 Methods: Financial Valuation

Thomer et al (2025) financial valuation framework was used on the data volume that was provided either by the database maintainers (see Curator Review datasheet) or the most recent version of the database as of June, 2025.

The rationale of the Thomer et al (2025) valuation centers around the cost to replace the data if only labour, expertise, and institutional overhead were required. The rationale also assumes that the data can be collected again, that the sites are still accessible, and equal quality specimens can be obtained. Within paleontology and earth sciences, this is often not the case.

We elected to focus on only two of the options: sample value ($150 USD) and site value ($3000 USD). See *Sheet 2* of Curator Review.

Additional costings (not listed in Thomer et al 2025) for data hosting, hiring database maintainters and developers, and curatorial labour were not included in the valuation.

6 Relevant links

Government of South Australia, 2017, "Fact sheet 3: Language list by country and place" in https://www.dpc.sa.gov.au/responsibilities/multicultural-affairs/policy/interpreting-and-translating-policy "https://www.dpc.sa.gov.au/__data/assets/pdf_file/0017/140516/Fact-sheet-3-Language-list-by-country-place.pdf"

Aggregator websites:

1. **https://www.webofscience.com/wos/woscc/basic-search**
2. **https://scholar.google.com/**

7. Citations

Kocsis, Á.T., Reddin, C.J., Alroy, J. and Kiessling, W. (2019) The R package divDyn for quantifying diversity dynamics using fossil sampling data. Methods in Ecology and Evolution, 10(5), pp.735-743.

National Academies of Sciences, (2018).*Open Science by Design: Realizing a Vision for 21st Century Research*. 1–232

Thomer, A., Williams, J., Goring, S., & Blois, J. (2025) The Valuable, Vulnerable, Long Tail of Earth Science Databases. Eos, 106. https://doi.org/10.1029/2025EO250107
