# Supplementary material for: The billion-dollar case for sustaining palaeontology’s digital databases
Source: Nat Ecol Evol. 2026 Feb 10;10(3):594–605. doi: 10.1038/s41559-026-02985-8 (PMC12971485; doi:10.1038/s41559-026-02985-8)
Supplement: Supplementary file 2 — Reporting Summary [file 41559_2026_2985_MOESM2_ESM.pdf]

nature portfolio

Corresponding author(s): Dowding Elizabeth M

Last updated by author(s): 2025/11/12

Reporting Summary

Nature Portfolio wishes to improve the reproducibility of the work that we publish. This form provides structure for consistency and transparency in reporting. For further information on Nature Portfolio policies, see our Editorial Policies and the Editorial Policy Checklist.

Statistics

For all statistical analyses, confirm that the following items are present in the figure legend, table legend, main text, or Methods section.

n/a	Confirmed	
☐	☒	The exact sample size (*n*) for each experimental group/condition, given as a discrete number and unit of measurement
☐	☒	A statement on whether measurements were taken from distinct samples or whether the same sample was measured repeatedly
☐	☒	The statistical test(s) used AND whether they are one- or two-sided<br>*Only common tests should be described solely by name; describe more complex techniques in the Methods section.*
☒	☐	A description of all covariates tested
☐	☒	A description of any assumptions or corrections, such as tests of normality and adjustment for multiple comparisons
☐	☒	A full description of the statistical parameters including central tendency (e.g. means) or other basic estimates (e.g. regression coefficient) AND variation (e.g. standard deviation) or associated estimates of uncertainty (e.g. confidence intervals)
☒	☐	For null hypothesis testing, the test statistic (e.g. *F*, *t*, *r*) with confidence intervals, effect sizes, degrees of freedom and *P* value noted<br>*Give P values as exact values whenever suitable.*
☒	☐	For Bayesian analysis, information on the choice of priors and Markov chain Monte Carlo settings
☒	☐	For hierarchical and complex designs, identification of the appropriate level for tests and full reporting of outcomes
☒	☐	Estimates of effect sizes (e.g. Cohen's *d*, Pearson's *r*), indicating how they were calculated

Our web collection on statistics for biologists contains articles on many of the points above.

Software and code

Policy information about availability of computer code

Data collection	Curator review data was collected from a survey of the author team and involved reporting information as of Nov 2024 about the back-end development, data volume, and funding of the databases the author represented. Publication products was supplied by the curators who collated formal citations of their work (the database publication) in other published literature; these were then assigned keywords on broad topics within palaeontology. Database diversity dynamics data was collected from a web survey of databases first publication, most recent publication, and whether the database was still actively available and maintained. These were conducted in the last quarter of 2024 and the first of 2025.<br>Data and code are available at CodeOcean, Zenodo and GIThub [https://github.com/dowdingem/IRAL]. The study has no restriction on data availability.<br><br>All data, code and supplementary material:<br>Dowding, E. M., et al. Fossils for Future and the billion dollar case for palaeontology's digital infrastructure. [Dataset] Zenodo. (2025). https://doi.org/10.5281/zenodo.17828000<br><br>Code and relevant data:<br>Dowding, E. M., et al. Fossils for Future: the billion dollar case for palaeontology's digital infrastructure. [Codebase] CodeOcean. (2025) https://doi.org/10.24433/CO.1586965.v1
Data analysis	Analysis was run in R v4.5.0 using the DivDyn Rpackage (Kocsis et al 2019) which contains metrics for richness, origination, extinction, and range through diversity amongst others. The information is available on CodeOcean and the GIThub links provided. In the time series analyses,

the durations of active and recent <5 years old) databases were extended into the future and the analytical frame was truncated at 2024 to address edge effects.

For manuscripts utilizing custom algorithms or software that are central to the research but not yet described in published literature, software must be made available to editors and reviewers. We strongly encourage code deposition in a community repository (e.g. GitHub). See the Nature Portfolio guidelines for submitting code & software for further information.

Data

Policy information about availability of data

All manuscripts must include a data availability statement. This statement should provide the following information, where applicable:

- Accession codes, unique identifiers, or web links for publicly available datasets
- A description of any restrictions on data availability
- For clinical datasets or third party data, please ensure that the statement adheres to our policy

All data generated for the author survey, publication, products, and analysis are available within the Supplementary Material and the stable Zenodo repository Dowding, E. M., et al. Fossils for Future and the billion dollar case for palaeontology's digital infrastructure. [Dataset] Zenodo. (2025). https://doi.org/10.5281/zenodo.17828000
All code and required data are available through CodeOcean. All analyses were conducted using publicly available R packages, and the links have been provided in Methods.

Research involving human participants, their data, or biological material

Policy information about studies with human participants or human data. See also policy information about sex, gender (identity/presentation), and sexual orientation and race, ethnicity and racism.

Reporting on sex and gender	*Use the terms sex (biological attribute) and gender (shaped by social and cultural circumstances) carefully in order to avoid confusing both terms. Indicate if findings apply to only one sex or gender; describe whether sex and gender were considered in study design; whether sex and/or gender was determined based on self-reporting or assigned and methods used. Provide in the source data disaggregated sex and gender data, where this information has been collected, and if consent has been obtained for sharing of individual-level data; provide overall numbers in this Reporting Summary. Please state if this information has not been collected. Report sex- and gender-based analyses where performed, justify reasons for lack of sex- and gender-based analysis.*
Reporting on race, ethnicity, or other socially relevant groupings	*Please specify the socially constructed or socially relevant categorization variable(s) used in your manuscript and explain why they were used. Please note that such variables should not be used as proxies for other socially constructed/relevant variables (for example, race or ethnicity should not be used as a proxy for socioeconomic status). Provide clear definitions of the relevant terms used, how they were provided (by the participants/respondents, the researchers, or third parties), and the method(s) used to classify people into the different categories (e.g. self-report, census or administrative data, social media data, etc.) Please provide details about how you controlled for confounding variables in your analyses.*
Population characteristics	*Describe the covariate-relevant population characteristics of the human research participants (e.g. age, genotypic information, past and current diagnosis and treatment categories). If you filled out the behavioural & social sciences study design questions and have nothing to add here, write "See above."*
Recruitment	*Describe how participants were recruited. Outline any potential self-selection bias or other biases that may be present and how these are likely to impact results.*
Ethics oversight	*Identify the organization(s) that approved the study protocol.*

Note that full information on the approval of the study protocol must also be provided in the manuscript.

Field-specific reporting

Please select the one below that is the best fit for your research. If you are not sure, read the appropriate sections before making your selection.

☐ Life sciences ☐ Behavioural & social sciences ☒ Ecological, evolutionary & environmental sciences

For a reference copy of the document with all sections, see nature.com/documents/nr-reporting-summary-flat.pdf

Ecological, evolutionary & environmental sciences study design

All studies must disclose on these points even when the disclosure is negative.

Study description	The study focuses on the diversity trends of community run Earth science databases, centred around Palaeontology. The study is both qualitative and quantitative, descriptions of the databases both in terms of history, publication product, and technical development were provided by the curators through survey of the author team. Quantitative analysis of the diversity dynamics (richness, origination, cumulative sum, and extinction) were run using first and last appearance data of databases that were open access and community run. Both were completed to show the diversity of databases, both in terms of their proliferation, but also

	into terms of the variation in their volume, and technical development (back end construction). These together for the basis for conclusions and recommendations.
Research sample	Open access data bases were determined by both self description and the ability of a general member of the public to gain access to the data on their own or by request to the data administrator. Community run databases were identified by negative conditions, i.e. by not being managed by industry or government.
Sampling strategy	No sample size estimates were required as the analysis focuses on trends in time series and does no significance testing. Sampling strategy was by survey of the authorship team who represent the community run databases, and the diversity dynamics was by consistent search of free online aggregators using search terms in languages with the broadest geographic spread.
Data collection	Data was collected by Dowding using surveys which consisted both of a questionnaire and a spreadsheet for database curators to enter information about the database they maintain.
Timing and spatial scale	The information on databases first appearance (publication) and last point of activity (update publication or statement) was from the first appearance of large scale compilations that were published to 2024. Databases that fit the criteria of being open access and community run globally were included.
Data exclusions	Databases that were not open access or were governmental/industry maintained were excluded. Failing either or both of these categories resulted in exclusion from analysis.
Reproducibility	The data survey techniques are available in the supplementary information in addition to the raw data, clean data, and code. R ver. 4.5.0
Randomization	N/A
Blinding	Blinding was not required as there are no experimental constraints and information from the curators were self reported and they are named authors.

Did the study involve field work? ☐ Yes ☒ No

Reporting for specific materials, systems and methods

We require information from authors about some types of materials, experimental systems and methods used in many studies. Here, indicate whether each material, system or method listed is relevant to your study. If you are not sure if a list item applies to your research, read the appropriate section before selecting a response.

Materials & experimental systems

n/a	Involved in the study	
☒	☐	Antibodies
☒	☐	Eukaryotic cell lines
☒	☐	Palaeontology and archaeology
☒	☐	Animals and other organisms
☒	☐	Clinical data
☒	☐	Dual use research of concern
☒	☐	Plants

Methods

n/a	Involved in the study	
☒	☐	ChIP-seq
☒	☐	Flow cytometry
☒	☐	MRI-based neuroimaging

Plants

Seed stocks	*Report on the source of all seed stocks or other plant material used. If applicable, state the seed stock centre and catalogue number. If plant specimens were collected from the field, describe the collection location, date and sampling procedures.*
Novel plant genotypes	*Describe the methods by which all novel plant genotypes were produced. This includes those generated by transgenic approaches, gene editing, chemical/radiation-based mutagenesis and hybridization. For transgenic lines, describe the transformation method, the number of independent lines analyzed and the generation upon which experiments were performed. For gene-edited lines, describe the editor used, the endogenous sequence targeted for editing, the targeting guide RNA sequence (if applicable) and how the editor was applied.*
Authentication	*Describe any authentication procedures for each seed stock used or novel genotype generated. Describe any experiments used to assess the effect of a mutation and, where applicable, how potential secondary effects (e.g. second site T-DNA insertions, mosiacism, off-target gene editing) were examined.*
